# Mitochondrial Homeostasis in Pancreatic β Cell Function: Mechanisms and Therapeutic Targets for Diabetes

**DOI:** 10.1111/1753-0407.70228

**Published:** 2026-04-29

**Authors:** Ruihan Li, Bingqian Zhang, Yuxin Zhang, Yang Yang, Yuting Lu, Jingya Li

**Affiliations:** ^1^ The First Clinical Medical College, Nanjing University of Chinese Medicine Nanjing China; ^2^ State Key Laboratory of Drug Research, the National Center for Drug Screening, Shanghai Institute of Materia Medica, Chinese Academy of Sciences Shanghai China; ^3^ University of Chinese Academy of Sciences Beijing China; ^4^ School of Pharmaceutical Science and Technology, Hangzhou Institute for Advanced Study, University of Chinese Academy of Sciences Hangzhou China

**Keywords:** apoptosis pathway, mitochondrial homeostasis, mitochondrial quality control, organelle communication, pancreatic β cell dysfunction

## Abstract

Mitochondrial homeostasis is essential for pancreatic β cell function, and its disruption underlies diabetes pathogenesis. Chronic hyperglycemia, lipotoxicity, and inflammation impair mitochondrial quality control (MQC), leading to β cell dysfunction, oxidative stress, and apoptosis. Mitochondria‐organelle interactions, particularly with the endoplasmic reticulum (ER), lysosomes, and Golgi apparatus, further exacerbate β cell dysfunction by disrupting calcium signaling and metabolic coordination. Emerging potential therapies, such as DRAK2 inhibitors and metabolic reprogramming agents, show promise in preserving MQC and β cell function. However, clinical validation is needed. This review highlights mitochondrial dysfunction as a central driver of diabetes and underscores the potential of mitochondrial‐targeted strategies for therapeutic intervention.

## Highlights

- Mitochondrial dysfunction induced by metabolic stress compromises MQC, causing oxidative damage and β cell failure via impaired mitochondrial dynamics, mitophagy, and mitochondria‐associated membrane (MAM) signaling.
- Novel potential therapeutic strategies targeting autophagy‐related pathways (DRAK2 inhibitors), metabolic regulators (e.g., HDAC inhibitors), and inter‐organelle communication have shown potential in preserving β cell function.
- Dysregulation of mitochondria‐organelle interactions involving the endoplasmic reticulum, lysosomes, and Golgi apparatus exacerbates diabetes progression by disrupting calcium homeostasis, impairing mitochondrial turnover, and interfering with insulin trafficking.

## Graphical abstract

This review highlights mitochondrial dysfunction as a central driver of pancreatic β cell failure in diabetes, caused by disrupted mitochondrial quality control (MQC), oxidative stress, and impaired organelle communication. Emerging therapies, such as DRAK2 inhibitors and metabolic reprogramming agents, show promise in restoring β cell function by targeting mitochondrial homeostasis, offering potential new treatments for diabetes.

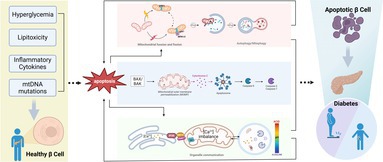

## 1 Introduction

Diabetes mellitus constitutes a highly prevalent metabolic disorder that poses a substantial threat to global public health. It is primarily characterized by chronic hyperglycemia and impaired insulin secretion or action [1]. According to the 11th Edition of the IDF Diabetes Atlas (2025), approximately 643 million individuals worldwide were living with diabetes in 2024, affecting one in nine adults aged 20–79 years (a total of 589 million cases within this age group). Projections indicate that this number will increase to 853 million by 2050, representing approximately one‐eighth of the global population [2]. Notably, data from 2023 revealed that only 4.4% of Chinese patients with diabetes achieve comprehensive treatment compliance [3], underscoring the significant challenges associated with effective disease management. Pancreatic β cells, as the sole source of insulin secretion, experience functional impairment, which serves as the fundamental pathophysiological basis for diabetes development [4]. Mitochondria, functioning as the key metabolic sensors and ATP producers, regulate the coupling of glucose oxidation to insulin exocytosis, thereby governing glucose‐stimulated insulin secretion (GSIS) in pancreatic β cells [5].

In recent years, mitochondrial dysfunction has been increasingly acknowledged as a pivotal contributor to diabetes pathogenesis. Multiple pathological mechanisms, including aberrant apoptosis activation [6], disrupted mitochondrial dynamics [5], excessive oxidative stress [7], impaired autophagy [8], and dysregulated inter‐organelle communication signaling [9, 10], have been conclusively demonstrated to induce pancreatic β cell apoptosis and compromise insulin secretion. Mitochondria play a critical role in regulating GSIS in β cells primarily through the production of ATP derived from oxidative phosphorylation (OXPHOS) [11]. Maternally inherited mitochondrial DNA (mtDNA) mutations, such as m.3243A>G, cause mitochondrial diabetes mellitus, which is characterized by premature β cell failure and insulin deficiency due to impaired OXPHOS [12]. Under physiological conditions, mitochondria homeostasis is maintained through three key mechanisms, (1) the dynamic balance of fusion and fission regulated by proteins such as DRP1 and MFN2 [5], (2) selective degradation of damaged components via the mitophagy pathway [13], and (3) coordination calcium signaling with endoplasmic reticulum (ER) [14]. Under diabetic conditions, chronic exposure to metabolic stressors, including hyperglycemia [15], lipotoxicity [16], and pro‐inflammatory cytokines [17], disrupts mitochondrial quality control (MQC) systems, activates the caspase cascade, and ultimately triggers β cell apoptosis. Mitochondrial homeostasis is extremely important to pancreatic β cell function.

**FIGURE 1:**
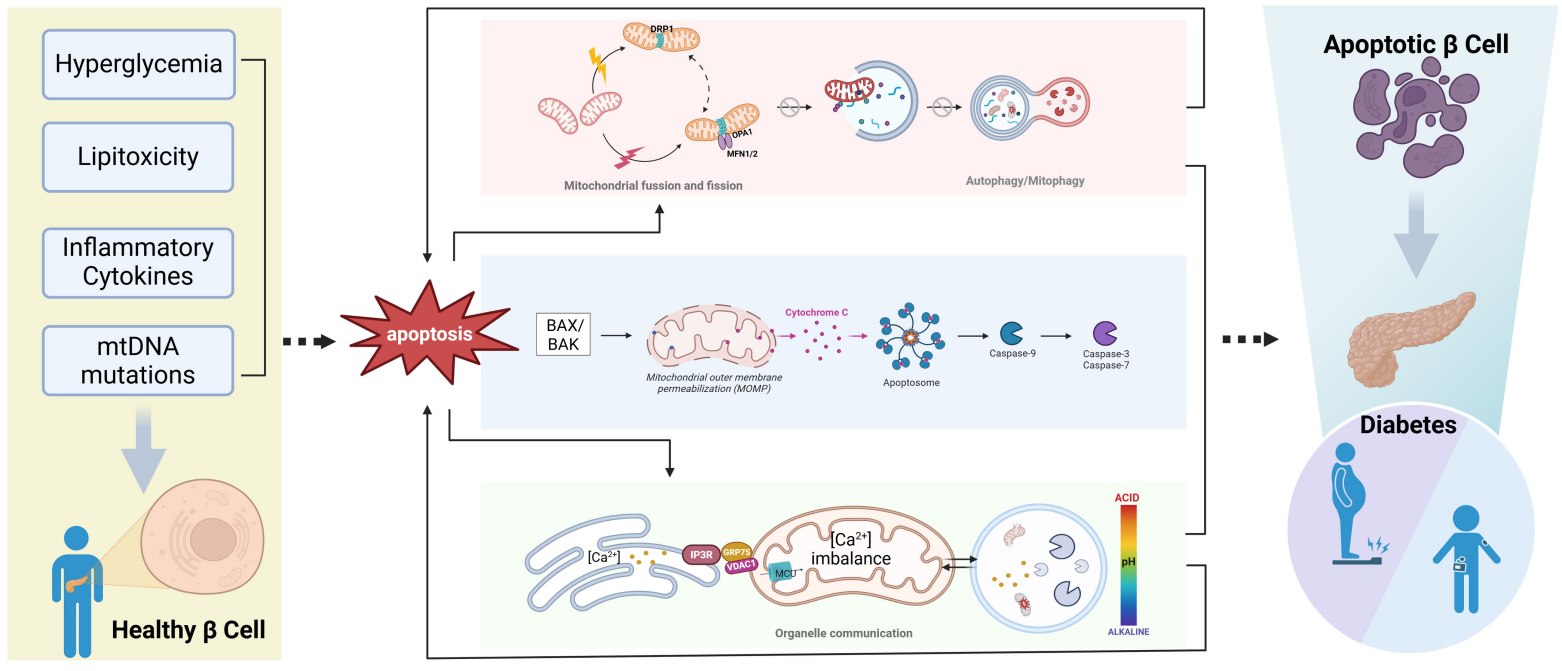
Mitochondrial homeostasis and pancreatic β cell dysfunction in diabetes. β cell mitochondria homeostasis and integrity are impaired under stress conditions such as hyperglycemia, lipotoxicity, inflammatory cytokines stimulation, and mtDNA mutation. An imbalance between mitochondrial fusion and fission, combined with impaired mitophagy, disrupts the mitochondrial quality control system. Interruption of signaling pathways further impairs communication between mitochondria and other organelles, including the endoplasmic reticulum and lysosomes, thereby amplifying cellular stress responses. This dysfunction promotes the activation of apoptotic signaling cascades, such as the release of cytochrome C via the BAX/BAK pathway, followed by Caspase‐3 and Caspase‐7 activation, which accelerates β cell loss and contributes to the progression of diabetes. These findings highlight the important role of mitochondrial dysfunction in the pathogenesis of diabetes. Furthermore, evidence from diabetes immunization studies suggests that immune‐mediated mechanisms may also contribute to β cell destruction.

Mitochondrial dysfunction profoundly impacts β cells through multiple mechanisms. This comprehensive review elucidates the mitochondrial regulatory axis in β cell dysfunction through three integrated dimensions, (i) apoptosis pathway modulation, (ii) MQC homeostasis, and (iii) organelle interaction networks (Figure 1). The integration of human genetics, metabolomics, and preclinical pharmacology has been demonstrated to identify actionable mitochondrial nodes for diabetes therapy, thereby bridging fundamental biology with translational opportunities.

## 2 Mitochondria and β Cell Apoptosis

**FIGURE 2:**
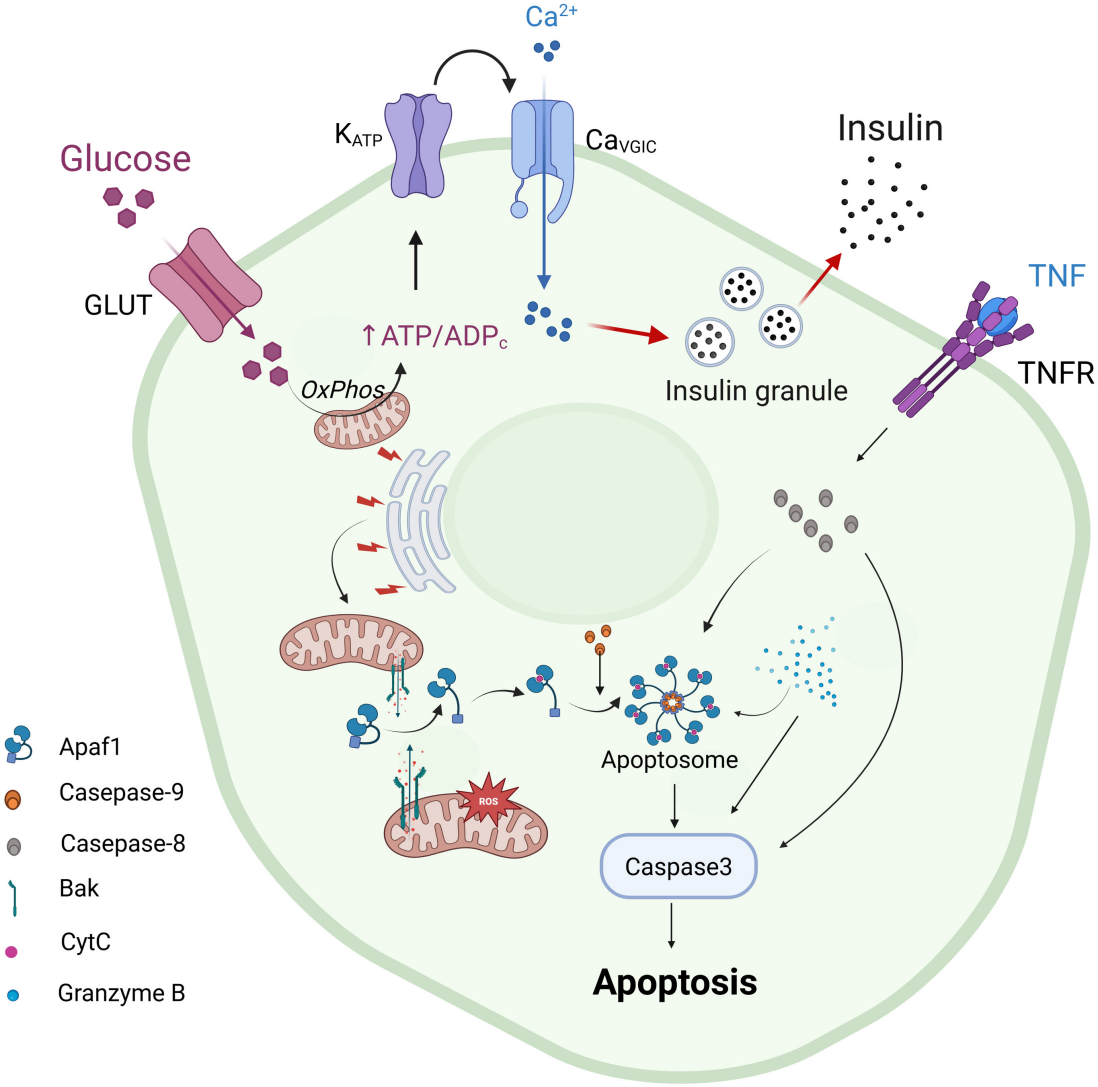
Mitochondria and β cell apoptosis. Four principal apoptotic pathways in eukaryotic cells: (1) the intrinsic (mitochondrial) pathway, (2) the extrinsic (death receptor) pathway, (3) the endoplasmic reticulum stress (ER‐stress) pathway, and (4) the perforin/granzyme pathway. In the mitochondrial pathway, apoptotic signaling regulates mitochondrial outer membrane permeabilization through the Bcl‐2 family of proteins, leading to the release of cytochrome C into the cytoplasm. Once released, cytochrome c binds to Apaf‐1 to form apoptosomes, which activate Caspase‐9. This subsequently triggers the activation of Caspase‐3, ultimately resulting in the cleavage of cellular substrates and the execution of programmed cell death. This pathway is mitochondria‐dependent and plays a critical role in regulating cell development and homeostasis. The remaining three apoptotic pathways—the death receptor (e.g., TNFR)‐mediated pathway, the ER‐stress pathway, and the granzyme (e.g., granzyme B) pathway—converge on the intrinsic mitochondrial pathway, modulating mitochondrial outer membrane permeabilization and ultimately activating Caspase‐3 to induce apoptotic cell death.

Current research has elucidated four principal apoptotic pathways in eukaryotic cells, (1) the intrinsic (mitochondrial) pathway, (2) the extrinsic (death receptor) pathway, (3) the endoplasmic reticulum stress (ER‐stress) pathway, and (4) the perforin/granzyme pathway (Figure 2). Mitochondria function as the central executioner in the intrinsic apoptotic pathway [6]. A pivotal event in this process is mitochondrial outer membrane permeabilization (MOMP) via the mitochondrial permeability transition pore (MPTP), which mediates the release of cytochrome C into the cytosol [6]. This critical step is tightly regulated by the Bcl‐2 protein family, where pro‐apoptotic Bax and anti‐apoptotic Bcl‐2 proteins compete to modulate MOMP induction [18]. An elevated Bax/Bcl‐2 ratio facilitate the activation of the caspase cascade, thereby driving apoptotic progression [18]. Furthermore, the tumor suppressor p53 and its transcriptional target p21 have been shown to amplify apoptosis through dual mechanisms, modulating the Bax/Bcl‐2 balance and/or directly activating caspase signaling pathways. Regarding the mitochondrial apoptosis pathway in pancreatic β cells, numerous research findings have been reported in recent years. In the early stages of research, it was demonstrated that palmitate induces the death of β cells through the mitochondrial apoptotic pathway. This process occurs by decreasing the expression of anti‐apoptotic proteins, such as Bcl‐xl and Bcl‐2 [19], while increasing the expression of pro‐apoptotic factors, such as death protein 5 (DP5) and p53 upregulated modulator of apoptosis (PUMA). These effects were observed in cloned, primary rat, and human β cells [19, 20]. Moreover, palmitate triggers mitochondrial DNA damage‐mediated apoptosis in INS‐1 cells through the excessive production of nitric oxide [21].

Farnesoid X Receptor (FXR/Nr1h4), a key bile acid receptor, plays an essential role in regulating cytochrome C oxidase subunit 6A2 (COX6A2)‐mediated mitochondrial apoptosis in pancreatic β cells under type 2 diabetic conditions. Specifically, the overexpression of COX6A2 enhances its interaction with voltage‐dependent anion channel 1 (VDAC1), thereby promoting the translocation of Bax to mitochondria, facilitating the release of cytochrome C from mitochondria into the cytoplasm, and ultimately inducing β cell apoptosis. FXR negatively regulates COX6A2 expression by inhibiting the binding of histone acetyltransferase p300 and by reducing histone H3 lysine 27 acetylation on the *Cox6a2* promoter. Consequently, FXR deficiency exacerbates β cell apoptosis in diabetic states [22].

Recent studies have demonstrated that the aggregation of human islet amyloid polypeptide (hIAPP) triggers the mitochondrial apoptotic pathway in pancreatic β cells. Specifically, hIAPP aggregation within β cells promotes the release of cytochrome C, activates Caspase‐9, and subsequently induces apoptosis. Notably, the suppression of Fas signaling results in a substantial reduction of apoptosis induced by extracellular hIAPP aggregates. In contrast, the inhibition of Bax/caspase‐9 activity leads to a modest attenuation of this effect. These findings underscore the significance of the mitochondrial apoptotic pathway in amyloid‐induced β cell death and suggest potential therapeutic strategies for mitigating intracellular amyloid β‐cytotoxicity by targeting the apoptotic function of cytochrome C [23].

The mitochondrial inner membrane protein MPV17 has been identified as a novel regulator of β cell apoptosis. Phenotypic analyses of diabetic mice and in vitro cultured β cells have demonstrated that MPV17 plays a critical role in promoting β cell apoptosis [24]. Notably, existing research indicates that MPV17 deficiency is associated with unfavorable outcomes, including a decrease in mtDNA abundance in hepatocytes, which can result in liver failure [25], as well as an enhancement of podocyte and neuronal apoptosis [26, 27, 28], and an exacerbation of cardiomyocyte ischemia–reperfusion injury [29]. Consequently, further investigation is necessary to elucidate the precise mechanisms by which MPV17 mediates β cell apoptosis [24].

In addition to proteins, recent studies have revealed that noncoding RNAs also play a critical role in modulating the mitochondrial apoptosis pathway in β cells. For instance, circRNA circGlis3, which is highly enriched in pancreatic islets and originates from an exon sequence of *Glis3*, has been shown to be significantly upregulated in both genetically obese and dietary obese mouse models. Mimicking this upregulation in high‐fat diet (HFD)‐fed mice and aged Lepr^*db/db*^ mice enhances insulin secretion and alleviates β cell apoptosis. Mechanistically, circGlis3 acts as a molecular sponge for miR‐124‐3p, thereby promoting insulin transcription and secretion. Additionally, its interaction with the pro‐apoptotic protein SCOTIN alleviates apoptosis through a Caspase‐3 inhibition‐dependent mechanism. In the context of sustained hyperglycemia, the expression of circGlis3 is observed to be induced by the splicing factor QKI. However, excessive stress and decompensation in β cells may lead to cytoplasmic accumulation of FUS, which sequesters free circGlis3, thereby reducing its abundance and impairing its ability to mitigate β cell apoptosis [30].

Among microRNAs, miR‐25 has been identified as a key regulator in IL‐1β‐induced β cell injury. Specifically, the miR‐25 sequences target genes *Neurod1* and *Mcl1*, leading to the downregulation of NEUROD1 and MCL1 protein expression. This subsequently promotes β cell apoptosis. The miR‐25/NEUROD1 axis mitigates β cell apoptosis through the transcriptional regulation of β cell‐specific genes. Conversely, the miR‐25/MCL1 axis induces β cell apoptosis via a Caspase‐3/PARP1‐dependent mechanism [31]. Additionally, research has demonstrated that miR‐344‐5p influences β cell apoptosis, apoptotic Caspase‐3/Bax signaling, and the downstream MAPK/ERK signaling pathways of the insulin receptor. It is noteworthy that the expression level of microRNA‐344‐5p is markedly reduced in rat pancreatic beta cells exposed to cholesterol and palmitate. Mechanistically, *Caveolin‐1* (*Cav1*) has been identified as a direct downstream target of miR‐344‐5p. Silencing *Cav1* partially reverses cholesterol‐induced β cell dysfunction and apoptosis. Collectively, these findings suggest that the miR‐344‐5p/*Cav1* axis regulates cholesterol‐induced β cell apoptosis and dysfunction through the involvement of apoptotic Caspase‐3/Bax signaling and MAPK/ERK signaling pathways [32]. In gestational diabetes mellitus (GDM), existing studies have demonstrated that the expression of microRNA‐770‐5p (miR‐770‐5p) is upregulated, while the expression of apoptosis inhibitory protein 1 (TRIAP1), a direct target of miR‐770‐5p, is correspondingly downregulated. miR‐770‐5p negatively regulates TRIAP1 expression in INS‐1 cells. Treatment with a miR‐770‐5p inhibitor promotes insulin secretion and increases total insulin content in INS‐1 cells, whereas TRIAP1‐siRNA markedly suppresses these effects. Moreover, the miR‐770‐5p inhibitor enhances INS‐1 cell proliferation and suppresses apoptosis by reducing the expression of Bax and apoptotic peptidase‐activating factor 1 (APAF1), while simultaneously increasing Bcl‐2 levels in INS1 cells. However, these effects are reversed upon treatment with TRIAP1‐siRNA. In summary, miR‐770‐5p serves as an important regulator of proliferation, apoptosis, and insulin secretion in pancreatic β cells through its targeting of TRIAP1. Dysregulation of miR‐770‐5p contributes to the pathogenesis of GDM by modulating the APAF1 signaling pathway [33].

DAP kinase‐related apoptosis‐inducing protein kinase 2 (DRAK2) constitutes one of five members of the death‐associated protein kinase (DAPK) family, which is involved in the regulation of cell apoptosis [34, 35, 36]. The pancreatic islets of *Drak2* transgenic mice exhibit increased susceptibility to apoptosis upon stimulation by inflammatory cytokines, mimicking the type 1 diabetes (T1D) microenvironment, thereby impairing insulin secretion capacity [37]. Treatment with free fatty acids (FFA) significantly upregulates DRAK2 expression in β cells, and pancreatic β cells from *Drak2* transgenic mice exhibit heightened vulnerability to FFA‐induced apoptosis [38]. These findings collectively suggest that DRAK2 upregulation is closely associated with pancreatic β cell apoptosis.

## 3 Mitochondrial Quality Control and β Cell Function

MQC constitutes a sophisticated network that monitors mitochondrial integrity and functions as an endogenous cellular protective mechanism essential for maintaining mitochondrial homeostasis and functionality [39]. MQC orchestrates mitochondrial homeostasis by coordinating key processes, including biogenesis, fission, fusion, proteolysis, and mitophagy [40].

**FIGURE 3:**
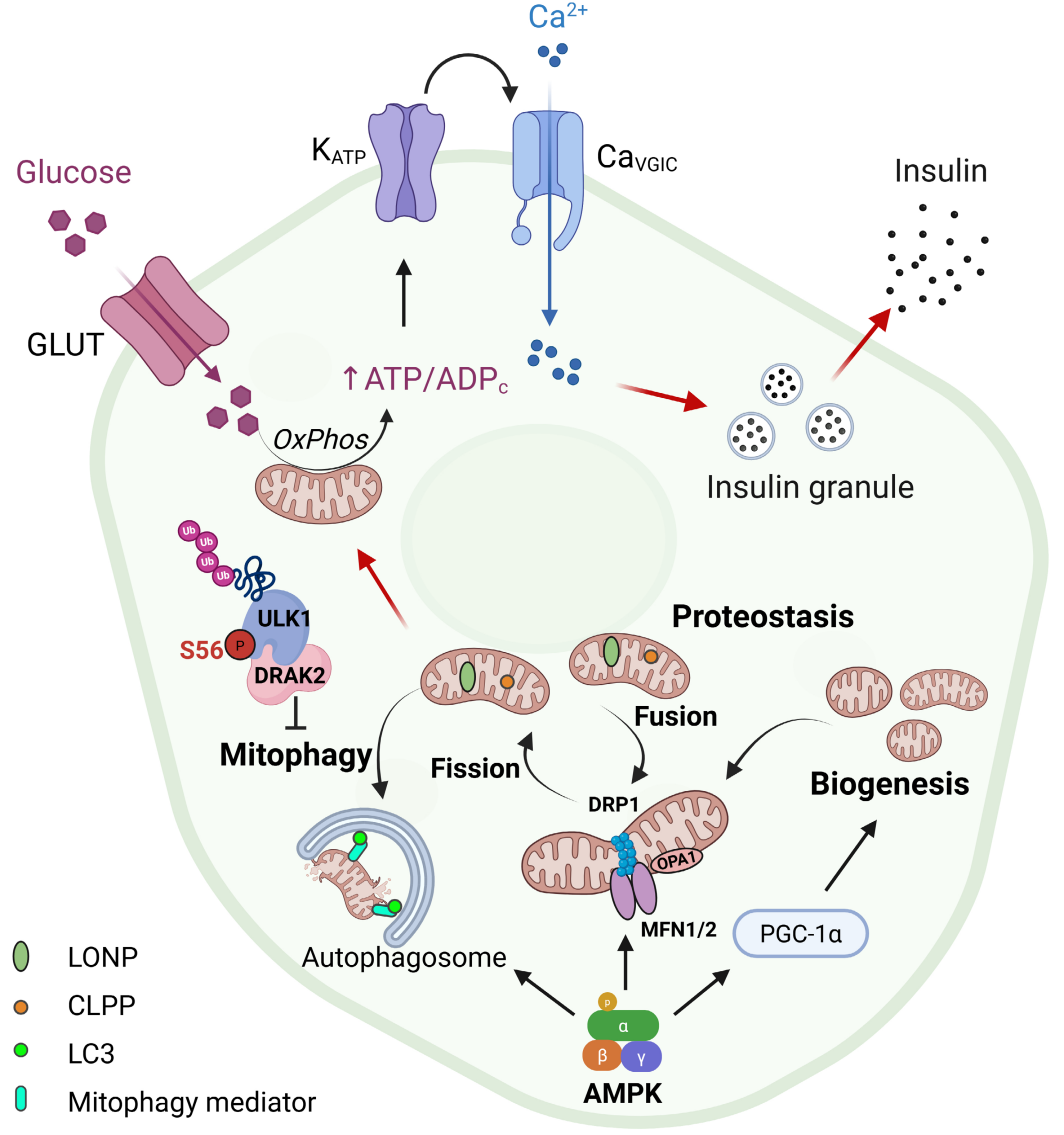
Mitochondrial quality control and β cell function. Glucose metabolism activates pathways such as GLUT‐mediated uptake and K_ATP_ channel regulation, which are modulated by ATP/ADP ratios, oxidative phosphorylation, and mitophagy. Calcium signaling serves as a central regulator of insulin secretion and mitochondrial function. AMPK, functioning as a key metabolic sensor, mediates mitochondrial dynamics, mitophagy, and biogenesis. DRAK2 enhances ULK1 degradation through phosphorylation at its Ser56 residue, consequently suppressing autophagy and diminishing mitochondrial quality. The proteases LONP and CLPP are emphasized for their essential roles in maintaining mitochondrial protein homeostasis, whereas LC3 and other mitophagy‐related factors facilitate the clearance of damaged mitochondria.

Under normal physiological conditions, mitochondrial function exhibits a progressive decline over time. To compensate for this decline, mitochondria undergo fusion to share internal components and sustain functionality. Subsequently, when mitochondrial dysfunction recurs, fission occurs to isolate and eliminate damaged mitochondria via autophagic pathways. Healthy mitochondria undergo fusion, while their damaged counterparts undergo degradation. These dynamic processes (fission, fusion, and mitophagy) collectively optimize mitochondrial performance, with biogenesis serving as a regenerative mechanism to replenish the mitochondrial pool (Figure 3).

### 3.1 Mitochondrial Biogenesis

Mitochondrial biogenesis represents a regenerative process that replaces aged or damaged mitochondria with newly synthesized, functional ones. This complex process is orchestrated by both mtDNA and nuclear DNA, encompassing a series of critical steps such as mtDNA replication, protein synthesis, membrane assembly, and network expansion [41]. Key regulators of this process include peroxisome proliferator‐activated receptor gamma coactivator 1‐alpha (PGC‐1α), nuclear respiratory factors 1/2 (NRF1/2), and mitochondrial transcription factor A (TFAM) [42]. PGC‐1α is modulated by cellular energy status, such as the AMP/ATP ratio, as well as sirtuin 1/3 (SIRT1/3)‐mediated deacetylation [43]. It enhances the interaction of NRF1/2 with the promoters of electron transport chain (ETC) genes and activates TFAM to promote mtDNA replication. The newly generated mitochondria via biogenesis enhance ATP production to meet metabolic demands in both physiological contexts, such as exercise and cold exposure, as well as in pathological conditions, including diabetic cardiomyopathy [44].

In pancreatic β cells, mitochondrial ATP production serves as the driving force for GSIS. Reduced expression of PGC‐1α compromises mitochondrial quality, ATP synthesis, and insulin secretion. In type 2 diabetes (T2D), the downregulation of PGC‐1α in β cells is closely associated with mitochondrial dysfunction [45]. Restoring PGC‐1α activity enhances mitochondrial biogenesis, thereby improving insulin secretion efficiency [46]. NRF2, another pivotal transcription factor, plays a critical role in maintaining neonatal redox balance, promoting mitochondrial biogenesis, and supporting β cell proliferation, thus preserving functional β cell mass under metabolic stress conditions [47]. The loss of mitochondrial transcription factor B1 (TFB1M) disrupts mitochondrial function and contributes to the pathogenesis of T2D [48]. Similarly, defects in ribosomal processing, such as the overexpression of dimethyladenosine transferase 1 homolog (DIMT1), an rRNA methyltransferase, in human T2D islets, exhibit a positive correlation with insulin mRNA levels but a negative correlation with insulin secretion. DIMT1 deficiency impairs protein synthesis, leading to mitochondrial dysfunction and defective insulin secretion, both of which are implicated in the development and progression of T2D [49].

### 3.2 Mitochondrial Dynamics

Mitochondria are highly dynamic organelles that undergo continuous cycles of fusion and fission, thereby remodeling their morphology, size, and distribution. This process is known as mitochondrial dynamics [44, 50]. The process of fission segregates dysfunctional mitochondria from healthy ones, while fusion enables the exchange of molecular components (e.g., DNA, proteins, and metabolites) between mitochondria, thereby restoring network integrity [51, 52]. The balanced interplay between fission and fusion maintains two distinct mitochondrial populations, with fragmented dysfunctional organelles and elongated interconnected networks, both of which are essential for cellular health [53].

#### 3.2.1 Mitochondrial Fusion

Fusion represents a protective mechanism wherein two mitochondria integrate their outer and inner membranes to form a single tubular structure [39, 54]. Outer mitochondrial membrane (OMM) fusion is mediated by mitofusin 1/2 (MFN1/2), while inner mitochondrial membrane (IMM) fusion requires optic atrophy 1 (OPA1). This process enables the exchange of mtDNA, phospholipids, ETC proteins, and tricarboxylic acid (TCA) cycle intermediates, thereby generating organelles with heterogeneous membrane potentials and diverse molecular pools [55].

MFN1/2, which are structurally similar GTPases [56], form homo‐ or heterodimers through their coiled‐coil domains [57]. The hydrolysis of GTP induces conformational changes that facilitate the fusion of adjacent OMMs. A complete knockout of *Mfn1/2* is embryonic lethality, highlighting their critical role in development. In contrast to the fusions of OMMs between two mitochondria, the fusion of IMMs occurs more rapidly and completely [54, 58]. In vivo, OPA1 exists in two forms, the long transmembrane form (l‐OPA1) which is connected to the IMM, and the short soluble form (s‐OPA1) which is generated by proteolytic cleavage at the S1 site within the inner membrane space. During the process of fusion, these two forms of OPA1 act as mediators, facilitating the fusion of IMMs between disparate mitochondria [59].

In T2D, the downregulation of MFN2 in β cells leads to mitochondrial fragmentation, cristae disruption, impaired ETC assembly, reduced ATP production, and defective insulin secretion [60]. Elevated levels of FFA suppress MFN2 expression, thereby promoting mitochondrial fission and inducing pancreatic β cell apoptosis. The activation of OMA1 enhances OPA1 cleavage, causing IMM fusion failure, cytochrome C release, and caspase‐dependent β cell apoptosis [61]. Research has indicated that in response to mitochondrial stress induction, there is an increase in both TNFα and OPA1 expression. Restoring intracellular mitochondrial dynamics by enhancing mitochondrial fusion and improving crista structure can improve respiratory chain efficiency. The TNFα‐NFĸB‐OPA1 signaling pathway plays a pivotal role in regulating mitochondrial integrity in both primary mouse pancreatic islets and insulin‐secreting MIN6 cells [62].

#### 3.2.2 Mitochondrial Fission

Fission fragments the tubular mitochondrial networks into smaller organelles, thereby facilitating mitophagy. The process includes DRP1 phosphorylation (activation) in response to membrane depolarization or damage, followed by the recruitment of DRP1 to the OMM via adaptors such as fission 1 (Fis1), mitochondrial fission factor (MFF), mitochondrial dynamic protein 49/51 (MiD49/51), and GTP‐dependent constriction mediated by DRP1 [63]. Mitochondrial fission isolates damaged mitochondria, ensures the maintenance energy production, and involves interactions with mitochondria‐associated membranes (MAMs) and ER in the regulation of DRP1 recruitment [64].

In β cells, hyperglycemia or oxidative stress activates PKC‐dependent DRP1, leads to mitochondrial fragmentation, reduced ATP levels, impaired GSIS, and reactive oxygen species (ROS) overproduction. This, in turn, causes mtDNA damage, ER‐stress, and ultimately, apoptosis [65, 66]. In contrast, studies have shown that β cell‐specific *Drp1* knockout mice exhibit elongated mitochondria, increased ATP production, and enhanced GSIS [67]. The DRP1 inhibitor Mdivi‐1 restores mitochondrial networks and normalizes insulin secretion in diabetic models [68]. Additionally, Fis1 and MiD51 regulate mitochondrial dynamics and insulin secretion [69, 70]. Physiologically, a dynamic equilibrium between mitochondrial fusion and fission is maintained to ensure proper mitochondrial function. Disruption of this balance impairs mitochondrial function and contributes to the pathogenesis of diseases such as diabetes [71].

### 3.3 Mitophagy

Mitophagy primarily achieves quality control by selectively degrading damaged or dysfunctional mitochondria. One of the most prominent mechanisms of mitophagy is the PINK1/Parkin pathway. Damaged mitochondria can also be cleared through receptor‐mediated pathways, including Bcl2 interacting protein 3‐like (BNIP3L/NIX) or FUN14 domain containing 1 (FUNDC1), as well as nonclassical autophagy that does not rely on ULK1 or ATG5 [72, 73, 74]. Beyond the established PINK1‐dependent and independent pathways, other nonclassical mechanisms exist for the clearance and recovery of mitochondria. A lysosome‐driven degradation pathway involving mitochondria‐derived vesicle has been confirmed, which responds rapidly when subjected to oxidative stress, eliminating damaged but undepolarized mitochondria [75, 76]. In addition, mitochondria can be expelled from cells via efflux mechanisms, forming extracellular vesicles that encapsulate the mitochondria, known as exospheres [77]. With the progression of research on both ubiquitin‐dependent and ubiquitin‐independent pathways of mitophagy, an expanding range of human diseases have been linked to mitochondrial dysfunction [74]. When mitochondria experience excessive stress or fail in quality control processes, cellular, tissue‐level, and organismal responses are elicited. Abnormal mitochondrial function has been implicated in metabolic disorders, immune‐inflammatory diseases, and neurological conditions [78].

In the classical PINK1/Parkin‐dependent pathway, PINK1 on healthy mitochondria is transported to the IMM through the translocase of the outer membrane and translocase of the inner membrane complexes. There, the protein is cleaved and degraded by the presenilin‐associated rhomboid‐like (PARL) protease, maintaining low basal levels [79]. Upon mitochondrial damage and loss of membrane potential, the transport of PINK1 is disrupted, leading to its accumulation on the OMM and the subsequent activation of its kinase activity. PINK1 phosphorylates Parkin and OMM proteins, thereby recruiting Parkin to mitochondria [79]. Subsequently, Parkin ubiquitinates MFN1/2 and VDAC1, tagging mitochondria for autophagic degradation [80]. The ubiquitinated signals are recognized by autophagy receptors such as optineurin (OPTN), nuclear dot protein 52 (NDP52), and sequestosome 1 (p62), which recruit LC3‐II‐labeled autophagosomes. These autophagosomes ultimately fuse with lysosomes to degrade mitochondria [81]. In the NIX/BNIP3 receptor‐mediated pathway, hypoxia or developmental signals induce the expression of these proteins [82]. Their LC3‐interacting region directly binds to LC3, enabling mitochondrial degradation without requiring ubiquitination [72].

Impaired mitophagy is a hallmark feature of diabetes [83]. Chronic hyperglycemia suppresses AMPK activity, disrupts the PINK1/Parkin pathway, and inhibits autophagic processes [84]. Activation of AMPK or inhibition of mTOR can enhance mitophagy, thereby protecting β cell function [85, 86, 87]. FFA induce mitochondrial dysfunction while suppressing the expression of the autophagy receptor p62, leading to impaired mitophagy [88, 89]. Studies have demonstrated that systemic *Parkin* knockout mice exhibit impaired glucose tolerance, β cell mitochondrial function, and turnover. Following streptozotocin‐induced β cell injury, GSIS is significantly diminished [90]. β cell‐specific *Parkin* knockout mice display increased mitochondrial fragmentation, reduced ATP levels, and impaired GSIS. However, certain studies indicate that β cell‐specific *Parkin* deletion under diet‐induced obesity does not affect glucose homeostasis, insulin secretion, or β cell mass [91, 92]. Under HFD, the expression of PINK1 in β cells is downregulated, leading to the blockade of mitophagy and impaired clearance of mitochondria with excessive ROS or mtDNA mutations. This process accelerates the progression of diabetes [93]. In pancreatic islets from patients with T2D, Parkin expression is decreased, levels of mitophagy markers such as LC3‐II are diminished, and damaged mitochondria release cytochrome C and apoptosis‐inducing factors. These events activate caspase pathways and trigger β cell apoptosis. Mutations in PINK1 or Parkin are associated with an elevated risk of early‐onset diabetes [94]. The deficiency of the tumor suppressor p53 prevents the development of diabetes in both streptozotocin‐induced type 1 diabetic mice and *db/db* mice with type 2 diabetes. Glucolipotoxicity induces the accumulation of p53 in β cells, where cytoplasmic p53 disrupts mitophagy via an inhibitory interaction with Parkin, subsequently inducing mitochondrial dysfunction [91]. Another critical component in the PINK1/Parkin pathway is Miro1, an OMM GTPase that serves as a docking site for Parkin translocation. Miro1 deficiency reduces Parkin translocation and impairs mitophagy [95]. In human T2D islets and *db/db* mouse islets, the expression of Miro1 is significantly reduced, and β cell‐specific *Miro1* knockout in mice exacerbates glucose dysregulation under diet‐induced obesity. The deficiency of Miro1 disrupts β cell mitophagy, resulting in impaired insulin secretion and mitochondrial dysfunction [95, 96]. Additionally, research has demonstrated that pancreas‐specific FUNDC1 overexpression alleviates HFD‐induced mitochondrial defects and ER‐stress [97]. FUNDC1 plays a pivotal role in regulating lipotoxicity‐driven mitochondrial abnormalities, apoptosis, and dysfunction in pancreatic β cells.

Recent research has revealed a significant upregulation of DRAK2 expression in the pancreatic islets of both T2D patients and nonhuman primate models [98]. DRAK2 directly phosphorylates ULK1 at serine‐56 (Ser^56^), promoting ULK1 degradation via the proteasome pathway in a Ser [49] phosphorylation‐dependent manner, consequently suppressing autophagy containing mitophagy and diminishing mitochondrial quality. This process impairs mitochondrial function and compromises GSIS [98]. Long‐term intraperitoneal (i.p.) administration of a DRAK2 inhibitor 22b [99] alleviates the pathological processes associated with T2D in murine disease models, particularly those dependent on the DRAK2‐ULK1 axis [98, 100]. Bicyclic polyprenylated acylphloroglucinol‐related meroterpenoids, identified as potent DRAK2 inhibitors from *Hypericum patulum* , can enhance GSIS in mouse pancreatic islets [101]. Sulfuretin derivatives isolated from natural products and exhibiting DRAK2 inhibitory activity, effectively protect against palmitate‐induced apoptosis in INS‐1E cells while preserving GSIS function of mouse pancreatic islets [102]. Moreover, sulfuretin has been shown to promote mitophagy and alleviates apoptosis in hepatic cells [103]. In addition, luteolin, a naturally occurring flavonoid with DRAK2 inhibitory activity, alleviates diabetic symptoms in *db/db* mice by promoting autophagy and ROS clearance [104]. Collectively, these findings suggest that DRAK2 has the potential to serve as a promising MQC regulator and a therapeutic target, particularly for addressing pancreatic β cell dysfunction associated with T2D.

### 3.4 Mitochondrial Proteostasis

Mitochondrial protein homeostasis denotes the dynamic equilibrium of protein folding, assembly, and degradation within mitochondria, which is maintained through the concerted regulation of molecular chaperones, protease systems, and the mitochondrial unfolded protein response [105]. The heat shock protein 60 (HSP60)/co‐chaperone (HSP10) complex, functioning as a molecular chaperone system, forms a cage‐like structure that facilitates the ATP‐dependent folding of newly synthesized or damaged proteins [106]. Mutations in HSP60 result in protein aggregation and mitochondrial dysfunction. Additionally, mtHSP70 plays a critical role in transmembrane transport and the folding of polypeptide chains, while synergistically collaborating with HSP60 to repair misfolded proteins [107].

Insufficient expression of HSP60 or mtHSP70 disrupts the assembly of respiratory chain complexes, diminishes ATP production, and impairs GSIS [108]. Elevated O‐GlcNAcylation of HSP60 interferes with its interaction with Bax, thereby contributing to pancreatic β cell apoptosis [109]. Notably, significant reductions in HSP60 and Lon protease expression within human pancreatic islets are associated with mitochondrial protein aggregation and β cell functional failure. In GDM, the downregulation of placental mitochondrial HSP60 correlates with impaired insulin secretion in maternal β cells [110]. Small‐molecule drugs, such as HSF1 activators, can upregulate HSP60/HSP70 levels to restore β cell protein folding capacity [111].

Proteolysis functions as another pivotal mitochondrial quality control mechanism, addressing oxidative stress, misfolded or damaged proteins, and defects in electron transport chains. Mitochondrial proteases are key components of this process. Notably, major matrix proteases, such as Lon protease homolog (LONP) and CLPP, play indispensable roles in maintaining mitochondrial protein folding homeostasis [112]. LONP1 enhances β cell survival and mitigates hyperglycemia by promoting mitochondrial protein folding [113]. LONP degrades oxidatively damaged or misfolded mitochondrial matrix proteins to preserve proteostasis [114], while the CLPXP complex mediates ATP‐dependent degradation of misassembled respiratory chain complex subunits within the mitochondrial matrix [114]. Mutations in these proteases have been associated with human genetic disorders. For example, LONP1 mutations cause CODAS syndrome [115], whereas CLPP mutations lead to Perrault syndrome [116]. Decreased Lon protease activity results in the accumulation of toxic proteins, such as misfolded SOD2, which triggers MPTP opening and cytochrome C release, thereby promoting apoptosis [117]. Natural compounds, including curcumin, alleviate β cell proteotoxicity by enhancing Lon protease activity [118]. Collectively, mitochondrial proteolysis is essential for sustaining mitochondrial function.

## 4 Mitochondria‐Organelle Communication and β Cell Function

**FIGURE 4:**
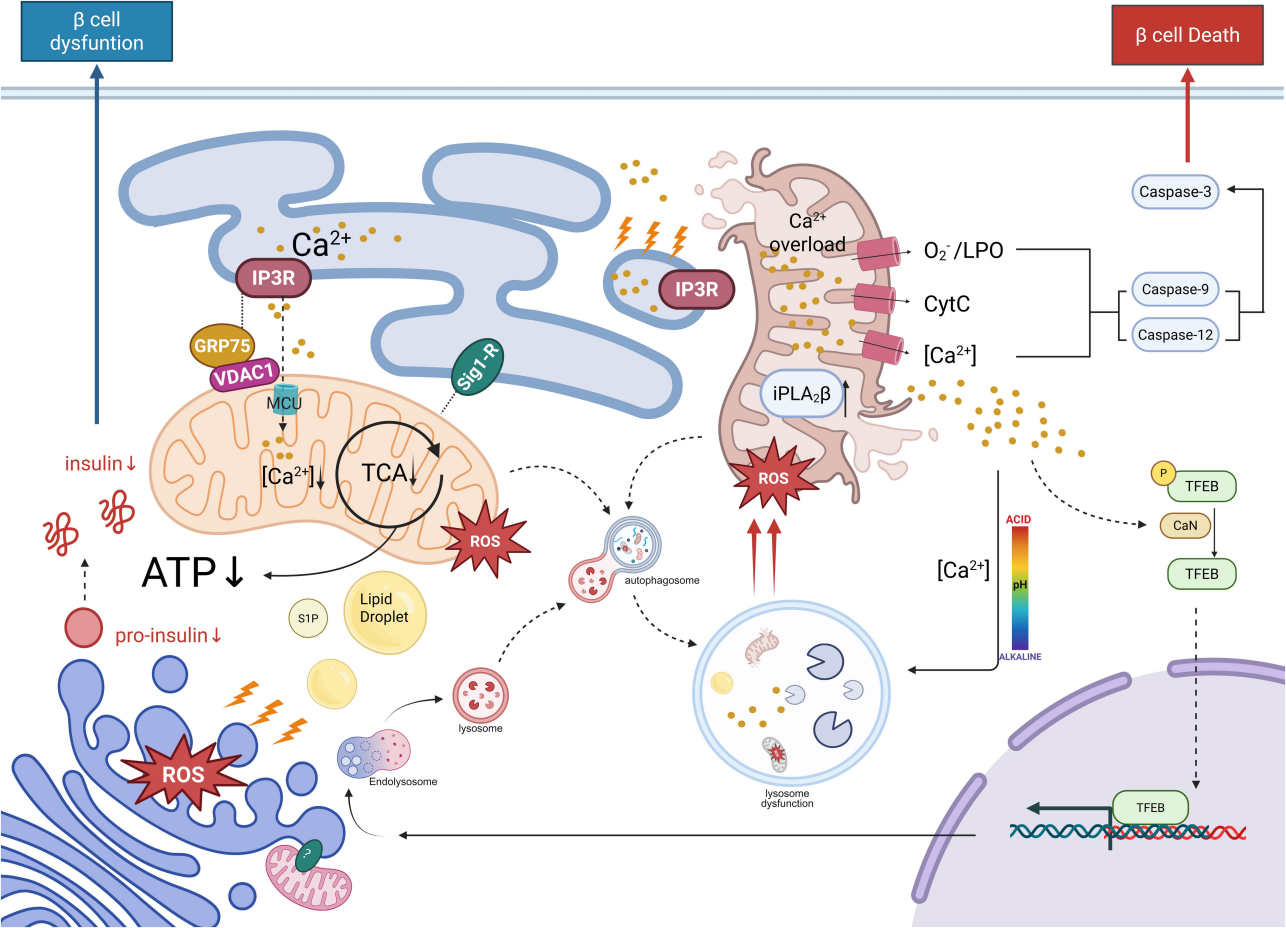
Mitochondria‐organelle communication and β cell function. In β cells, a network of inter‐organelle contacts facilitates metabolic coordination, vesicle trafficking, and stress responses. Mitochondria not only interact with the ER, but also with the Golgi apparatus, lysosomes, and lipid droplets to regulate key processes that support insulin biosynthesis and secretion. ER–mitochondria Ca^2+^ transfer stimulates mitochondrial ATP production, while the Golgi and lysosomes ensure proper proinsulin maturation and the degradation of misfolded proteins. Calcium signaling via IP3Rs bridges mitochondria–ER communication, which is also critical for autophagy and lysosomal acidification. Under glucolipotoxic conditions, excessive Ca^2+^ is transferred from the ER to mitochondria via the IP3R–GRP75–VDAC1 complex, leading to mitochondrial Ca^2+^ dyshomeostasis, TCA cycle dysregulation, and overproduction of ROS and iPLA_2_β. Simultaneously, defects in lysosomal acidification impair autophagic clearance. The release of Ca^2+^ activates TFEB and promotes its nuclear translocation through calcineurin‐mediated dephosphorylation, thereby altering the pattern of downstream gene transcription. Meanwhile, the Golgi apparatus and lipid droplet‐derived metabolites, such as S1P, further modulate mitochondrial homeostasis, disrupt proinsulin processing, and exacerbate organelle stress. Persistent oxidative and organelle stress signals ultimately activate Caspase‐3, ‐9, and ‐12, promoting β cell apoptosis. Collectively, these inter‐organelle disturbances impair insulin biosynthesis, secretion, and β cell survival, playing a central role in the pathogenesis of T2D.

The study of organelle interactions commenced with the establishment of vesicular transport theory [119, 120], and the concept of membrane contact sites emerged following two pivotal discoveries. Firstly, research conducted on lipid metabolism in the 1970s yielded the discovery of nonvesicular transport pathways [121, 122, 123]. Secondly, ultrastructural observations uncovered physical contact between the double membranes of mitochondria [124, 125]. In the early 1990s, scientists explored biochemical techniques to confirm the existence of the MAM and validated its role in mediating direct lipid transfer [126, 127, 128, 129]. As research progressed, the scientific community systematically unraveled the multifaceted mechanisms by which organelles exchange materials and information through membrane contact, structural fusion, and other processes. Beyond the ER, mitochondria in β cells also interact dynamically with other organelles, including lysosomes, the Golgi apparatus (GA), and lipid droplets (LD) [130]. These dynamic contact networks not only regulate organelle function but also influence overall cellular activity through cascade effects. Current studies have confirmed that imbalances in organelle interactions are closely linked to various pathological processes, including inflammation [131, 132], apoptosis [133, 134], autophagy [135], and disruptions in insulin production and secretion [136, 137]. The underlying mechanisms include the following: (1) ion regulation via bidirectional Ca^2+^ flux mediated by MAM [138], (2) signal transduction determining cellular fate through ROS and ATP output [133, 139], (3) metabolic synergy establishing directional transport channels for tricarboxylic acid cycle intermediates [140], and (4) lipid transfer enabling nonvesicular exchange of membrane components (Figure 4) [141, 142].

Existing evidence indicates that these connections are predominantly mediated by protein complexes rather than individual proteins. While microscopy has validated the spatial proximity of mitochondria to various organelles, the detailed characterization and functional analysis of associated protein complexes remain technically challenging owing to current limitations. This molecular redundancy in design underscores the fundamental role of the organelle interaction network in sustaining cellular life activities.

### 4.1 Mitochondria–ER Interaction

As the most extensive membrane system within cells, the ER orchestrates critical physiological processes through its heterogeneous subdomains. Ultrastructural analyses revealed that the ER can adopt tubular, sheet‐like, and highly organized “matrix”‐like configurations. Across its entire structure, the ER establishes distinct subdomains characterized by specific functions, unique lipid compositions, and specialized protein profiles [143, 144]. Within these subdomains, the ER supports protein folding and degradation via enzymatic activities, catalyzes the synthesis of phospholipids and cholesterol, and regulates Ca^2+^ flux and storage [145].

The majority of organelle interactions are centered around the ER. As two distinct organelles, the ER and mitochondria engage in dynamic and regulated physical interactions, thereby forming specialized microdomains. At the molecular level, these interactions are termed mitochondrial–ER contact sites or MAMs, where proteins such as VDAC‐GRP75‐IP3R1, PDZD8, Sig‐1R, and others mediate the establishment of physical connections [146, 147, 148].

MAM is a specialized subdomain composed of membrane components from both the ER and the OMM, harboring numerous cell‐specific molecular components that participate in complex formation [149, 150, 151]. Alterations in MAM composition and aberrant ER–mitochondrial interactions have been associated with various pathological conditions, necessitating the maintenance of a dynamic equilibrium. In T2D, an imbalance in these interactions causes β cell inflammation, apoptosis, and impaired metabolic function. Research findings have demonstrated that in β cells derived from T2D patients, there is a marked upregulation of inositol 1,4,5‐triphosphate receptor‐2 (IP3R2) at both protein and mRNA levels, while the expression of voltage‐dependent anion channel‐1 (VDAC‐1) is found to be downregulated. Additionally, in situ proximity assays reveal a significant reduction in the number of IP3R2‐VDAC‐1 complexes, indicating a diminished ER–mitochondrial interaction in human T2D β cells [147]. Palmitate treatment similarly reduces ER–mitochondrial interaction in Min6 cells [147]. Sigma‐1 receptor (Sig‐1R), an orphan receptor which is localized on the ER, aggregates at the MAM. Overexpression of Sig‐1R enhances the spatial proximity of the ER within 50 nm of mitochondria, thereby facilitating Ca^2+^ transport between these two organelles. Conversely, Sig‐1R deficiency exacerbates ER stress and mitochondrial dysfunction, ultimately contributing to palmitate‐induced β cell apoptosis [148, 150, 152, 153].

MAMs regulate the dynamic equilibrium of Ca^2+^ exchange between organelles by establishing calcium microdomains, thereby playing a central role in cellular Ca^2+^ homeostasis [154, 155]. Recent studies have further demonstrated that MAMs are involved in modulating mitochondrial dynamics [126], autophagy [156], ROS production [133], and mitochondrial‐mediated apoptosis and inflammation [157], Specifically, calcium signaling is crucial for mitochondrial energy metabolism in β cells. The key calcium ion channels on the ER, namely inositol trisphosphate receptors (IP3Rs or ITPRs) and ryanodine receptors (RyRs), mediate ER Ca^2+^ efflux. Specifically, RyR‐mediated Ca^2+^ flux is essential for maintaining the β cell ATP/ADP ratio, and inhibition of RyRs leads to impaired insulin secretion [158, 159]. Conversely, within a diabetic milieu, mitochondrial Ca^2+^ homeostasis becomes disrupted. The uptake of Ca^2+^ depends on the elevation of cytosolic Ca^2+^ [(Ca^2+^)_C_] and the release of ER Ca^2+^ [(Ca^2+^)_ER_], both of which are regulated by the two‐pore‐domain K(^+^) channel TALK‐1. The modulation of TALK‐1 activity influences β cell function by control of intracellular Ca^2+^ levels. Excessive Ca^2+^ overload in mitochondria induced by ER dysfunction sensitizes mitochondria to pro‐apoptotic stimuli, thereby promoting apoptosis through ROS production and cytochrome C release [160]. ER‐stress induces the accumulation of calcium‐dependent phospholipase A [2] [iPLA(2)β] in mitochondria, resulting in the opening of the mitochondrial permeability transition pore and a subsequent loss of mitochondrial membrane potential (MMP). This cascade activates Caspase‐12 and Caspase‐3 [161, 162]. Additionally, iPLA(2)β facilitates ceramide generation, which signals through multiple pathways, including the activation of extracellular signal‐regulated kinase 1 and 2 (ERK1/2), the downregulation of Per‐aromatic hydrocarbon receptor nuclear translocator (Arnt)‐single‐minded (Sim) kinase (PASK), the activation of Okadaic acid‐sensitive protein phosphatase 2A (PP2A), and the stimulation of NADPH oxidase to produce superoxide and lipid peroxides, thereby contributing to oxidative stress. Furthermore, iPLA(2)β promotes the mitochondrial release of cytochrome C into the cytoplasm, thereby accelerating apoptosis [161, 162].

Mitochondria, serving as another critical intracellular calcium reservoir, protect β cells by sequestering Ca^2+^ to a significant extent. In palmitate‐treated INS‐1E cells, the ER Ca^2+^ content decreases, whereas mitochondria and plasma membrane Ca^2+^ levels increase. This is accompanied by a reduction in MMP and ATP content. Furthermore, palmitate‐treated β cells exhibit a significant reduction in luminal Ca^2+^ content and impaired ER Ca^2+^ reuptake, resulting in a considerable reduction in insulin secretion capacity. Following ER Ca^2+^ depletion, extracellular Ca^2+^ influx elevates plasma membrane Ca^2+^ levels and activates adverse signaling pathways, ultimately causing cell death. The mitochondrial Ca^2+^ uniporter (MCU) represents the primary pathway for Ca^2+^ uptake into the mitochondrial matrix. The RNA interference targeting MCU expression can suppress mitochondrial superoxide production; however, it may concurrently exacerbate palmitate‐induced Ca^2+^ overload. This indicates that enhancing mitochondrial Ca^2+^ uptake by upregulation of MCU serves as a protective mechanism by which pancreatic β cells alleviate plasma membrane Ca^2+^ overload and its detrimental effects [163].

In addition to calcium, another important function concerning MAM is lipid metabolism and the transfer of lipid metabolites. Several proteins related to lipid trafficking and metabolism are involved in the formation of MAM [164, 165]. Since ER is the primary supplier of cellular lipid and provides mitochondria with lipids such as phosphatidic acid (PA), phosphatidylinositol (PI), phosphatidylserine (PS), phosphatidylcholine (PC), and sterols to synthesize mitochondria's own phospholipids including cardiolipin (CL), phosphatidylethanolamine (PE), and phosphatidylglycerol (PG), which are essential for mitochondrial membrane integrity and function [166]. Therefore, the destruction of MAM may cause perturbed phospholipid transportation and mitochondrial lipid oxidation dysfunction. Another example is Barth syndrome (BTHS) characterized by the deficiency of mitochondrial phospholipid: lysophospholipid transacylase and accumulation of the precursor of cardiolipin (CL), which is caused by mutations in the *Tafazzin* (*TAZ*) gene and will lead to defective formation of mitochondrial super‐complexes and reduced ATP production. Even though no phenotype of elevated blood glucose was observed in the *Taz* knockdown (*Taz*‐KD) mice due to adaptive mechanisms such as increased FGF‐21 secretion and compensatory proliferation of α cells, loss of *Taz* in the in vitro model of islets has been found to lead to defects in insulin secretion, accompanied by changes in lipid profile and mitochondrial metabolism impairment [167]. In addition, these changes also lead to a reduction in MAM contact sites and alterations in the mitochondrial lipid profile [168], which could further exacerbate β cell dysfunction. On the other hand, the enhancement of mitochondrial redox functions mitigates ER stress. Studies conducted on β cells that overexpress the fatty acid oxidase CPT1 have demonstrated that lipid toxicity‐induced apoptosis in INS‐1E cells is attenuated, accompanied by reduced expression of ER stress markers, such as p‐eIF2α and CHOP [169, 170]. These findings collectively emphasize the synergistic roles of mitochondrial and ER functions in maintaining cellular homeostasis.

### 4.2 Mitochondria Interaction With Lysosomes, the Golgi Apparatus, and Lipid Droplets

Simultaneous damage to both mitochondrial and lysosomal membranes can impair autophagic function. Cells that have been pretreated with 1,9‐dimethylmethane blue (DMMB) and subsequently exposed to irradiated induce specific organelle damage. At low concentrations (10 nM), DMMB selectively induces mitochondrial damage, thereby activating mitophagy. However, concurrent lysosomal membrane damage may result in incomplete activation of mitophagy, potentially leading to cell death [171]. Transcription factor EB (TFEB) serves as a central regulator of lysosomal biogenesis and autophagy‐related gene expression, with lysosomes playing an essential role in mitophagy. Mitochondrial or metabolic stress triggers mitophagy, which is mediated by lysosomal Ca^2+^ release, increased cytosolic [Ca^2+^], and subsequent TFEB activation. The ER‐lysosome Ca^2+^ reuptake pathway replenishes lysosomal Ca^2+^ release. The HFD‐diet also enhances mitophagy in pancreatic β cells, potentially as an adaptive response to metabolic stress. β cell‐specific *Tfeb* gene knockout reduces HFD‐induced mitophagy, accompanied by increased ROS levels, decreased mitochondrial cytochrome C oxidase activity, and reduced oxygen consumption. *Tfeb* knockout mice exhibit exacerbated glucose intolerance and impaired insulin secretion in response to HFD. These results indicate that lysosomal Ca^2+^ release, ER‐lysosome Ca^2+^ reuptake, and TFEB activation are critical for mitophagy under metabolic stress and for maintaining pancreatic β cell function [172, 173, 174].

Alterations in the redox state induced by cystine deposition in lysosomal storage diseases contribute to cellular dysfunction [140]. This rare condition, which arises due to a deficiency in the lysosomal cystine transporter (cystinosin), leads to excessive intra‐lysosomal cystine accumulation. A rat model of cystine storage disease was established using *Ctns*‐targeted siRNAs in cloned β cell lines. In this model, insulin secretion was diminished, and oxidative stress markers, including ROS, superoxide, and hydrogen peroxide, were increased. Additionally, elevated glutathione oxidation levels led to a decrease in the [reduced glutathione/oxidized glutathione] redox potential. These biochemical changes were accompanied by a reduction in MMP and an increase in cellular apoptosis [140].

Lipotoxicity induces a dose‐dependent lysosomal alkalinization and reduces mitochondrial turnover, resulting in an increase in mitochondrial mass. Restoring lysosomal acidity using lysosome‐targeted nanoparticles not only stimulated mitochondrial turnover, but also correlated with enhanced mitotic activity and recovery of mitochondrial function. Notably, reacidification significantly restored the enzymatic activity of citrate synthase and ATP content in INS‐1 cells. Moreover, nanoparticle‐mediated lysosomal reacidification effectively rescued the maximal respiratory capacity of mitochondria in both INS‐1 cells and primary mouse islets [175]. Collectively, these findings demonstrate that under lipid toxicity conditions, mitochondrial dysfunction is a downstream consequence of lysosomal alkalinization, and restoring lysosomal acidity is sufficient to ameliorate mitochondrial bioenergetic defects.

The GA is a critical organelle in secretory cells, such as β cells. However, the mechanisms by which the GA responds to stress in T2D remain incompletely elucidated. Studies have found that T2D β cells exhibit morphological alterations in the GA, including shortened and swollen Golgi cisternae, partial vesiculation of the Golgi, and expansion of the Golgi reservoir. These changes may potentially disrupt proinsulin trafficking [136, 176]. In T1D, pro‐inflammatory cytokines similarly induce complex structural and functional modifications in the β cell GA, including Golgi compression and loss of interconnected ribbon structures, leading to Golgi fragmentation [177]. Concurrently, the unique sensitivity of β cells to nitric oxide‐dependent mitochondrial inhibition results in specific alterations in the β cell GA, which are not observed in other cell types, including α‐cells [177]. RNA sequencing and microarray datasets derived from human islets of diabetic donors and human islets treated with the established GA stress (GA‐stress) inducer Brefeldin A (BFA) have revealed significant changes in key pathways associated with GA integrity, organization, and trafficking. Notably, genes linked to GA function, such as ATF3, ARF4, CREB3, and COG6, have been proposed as potential biomarkers for Golgi stress in β cells [178].

LDs are hypothesized to modulate β cell sensitivity to FFA, yet the underlying molecular mechanisms remain largely elusive. Intracellular sphingolipid‐1‐phosphate (S1P) metabolism significantly contributes to lipid toxicity‐induced β cell dysfunction. Specifically, both increased irreversible S1P degradation via S1P lyase overexpression and enhanced S1P cycling through S1P phosphatase 1 overexpression lead to reduced intracellular S1P concentrations, yet exert opposing effects on FFA sensitivity. Enhanced S1P cycling mitigates FFA‐induced Caspase‐3 activation by augmenting lipid storage capacity and alleviating oxidative stress. Conversely, increased S1P degradation restricts LD biogenesis, reduces LD content and size, and accelerates lipid phagocytosis [141], which is related to hydrogen peroxide production, mitochondrial fragmentation, functional impairment, and ER‐stress, thereby influencing β cell sensitivity to FFAs.

## 5 Mechanisms of Novel Therapeutic Approaches for Protecting β Cells by Regulating Mitochondrial Homeostasis

Therapies for diabetes, including metformin and SGLT‐2 inhibitors, have been shown to enhance β cell function by modulating mitochondrial dynamics. However, these therapies have been observed to be ineffective in preventing progressive β cell apoptosis or achieving functional regeneration. Emerging studies emphasize mitochondrial‐targeted approaches as potential therapeutic modalities, including (1) pharmacological inhibition of DRAK2‐ULK1 signaling to restore autophagic flux [98], (2) reversal of stress‐induced β cell dysfunction mediated by ISRIB [179, 180], and (3) metabolic reprogramming induced by small molecules (e.g., the HDAC inhibitor TH34) [181]. Studies have shown that the use of DRAK2 inhibitors can effectively alleviate pancreatic islet dysfunction caused by lipotoxicity [102, 104] and improve the symptoms of diabetic mice via autophagy/mitophagy promotion [98, 104]. The pharmacological inhibition of mitochondrial integrated stress response, using ISRIB, has been demonstrated to restore β cell mass, identity, and glucose homeostasis in mouse models [180]. This also suggests a strategy to counteract metabolic dysfunction. A small molecule named as TH34, which functions as an HDAC inhibitor, has been discovered to significantly enhance the maturation of human pluripotent stem cell‐derived β cells in vitro by lowering ceramide levels, improving insulin secretion, and restoring glucose responsiveness [181].

### 5.1 GLP‐1 Receptor Agonists and Mitochondrial Homeostasis

GLP‐1 receptor agonists (GLP1‐RAs) have a significant regulatory effect on the mitochondrial homeostasis of pancreatic β cells. This mechanism is closely related to their ability to improve β cell function, promote insulin secretion, and exhibit anti‐apoptotic effects. Semaglutide improves pancreatic β cell mitochondrial function by enhancing METTL14 expression, which promotes m6A methylation and upregulates PDX1, a key transcription factor for β cell development and function. This leads to improved mitochondrial autophagy via the Parkin pathway, reducing damage from lipotoxicity and oxidative stress. Additionally, Semaglutide modulates gut microbiota and increases short‐chain fatty acids (SCFAs), indirectly supporting β cell health. These combined effects restore mitochondrial integrity and function, alleviating β cell dysfunction in T2D [182]. Exendin‐4, as the main component of the Exenatide, effectively protects Min6 cells from ROS induced apoptosis by preserving mitochondrial function [183]. Liraglutide counters glucolipotoxicity by enhancing mitophagy, restoring mitochondrial bioenergetics, and reducing oxidative stress, thereby preserving RINm5F cell survival and function [184]. Dulaglutide was successful used in treating mitochondrial diabetes, specifically in a patient with maternally inherited diabetes and deafness caused by the m.3243A>G mutation in the *MT‐TL1* gene. The efficacy of Dulaglutide treatment with mitochondrial diabetes mellitus patients partially due to a reduced mitochondrial damage in β cells [185].

### 5.2 Glucokinase Activator and Mitochondrial Integrity

The glucokinase activator YH‐GKA positively impacts mitochondrial function in INS‐1 cells, particularly under glucotoxic conditions. The mechanism of YH‐GKA in detail mainly consists of several aspects: (1) restored cellular ATP levels, which were reduced by high glucose exposure, indicating improved mitochondrial energy production; (2) mitigated the disruption of MMP caused by glucotoxicity, as evidenced by reduced JC‐1 green fluorescence; (3) preserved the activity of citrate synthase, a critical enzyme in the TCA cycle, suggesting enhanced mitochondrial metabolic function; (4) upregulated genes related to mitochondrial function (e.g., citrate synthase, TFAM) and downregulated PGC‐1α, which is linked to inefficient ATP production in β cells; and (5) increased Bcl‐2 and decreased Bax protein levels, while enhancing the interaction between glucokinase and VDAC on mitochondria. This interaction is crucial for maintaining mitochondrial integrity and preventing apoptosis. Overall, YH‐GKA ameliorated glucotoxicity‐induced mitochondrial dysfunction, thereby promoting β cell survival and function [186].

### 5.3 Nanomedicines Targeting β Cell Mitochondrial Function

Research has demonstrated that the utilization of biodegradable polyesters delivered via nanoparticles (TFSA NPs) can facilitate β cell targeting and enhance β cell mitochondrial activity by restoring lysosomal acidity and autophagy function. The experimental findings suggest that these nanoparticles have the potential to ameliorate palmitic acid‐induced damage to INS‐1 cells and restore pancreatic function in HFD mice [187].

A newly developed customized nanomedicine, designated Mito‐G, has been developed with the capacity to sequentially target the islets and the mitochondria of β cells. This negatively charged ultra‐small nanomedicine has been synthesized by the polymerization of the potent UCP2 inhibitor ginkgoflavin and glycine. It has been demonstrated to be capable of specifically reaching β cells and exhibiting natural mitochondrial targeting. By specifically inhibiting β cell UCP2 activity and suppressing the STING pathway, this nanomedicine effectively eliminates inflammation cycles in β cells. It has also been demonstrated to alleviate endoplasmic reticulum stress in pancreatic β cells, thereby protecting their ER structure and function. This process ultimately leads to the restoration of mitochondrial function and, consequently, the preservation of β cells [188].

The effects mentioned above highlight therapeutic potential for diabetes by targeting pancreatic β cell mitochondrial pathways. The outstanding performances of nanoapproaches suggest the potential for novel methods of protecting mitochondria to be promising strategies. It also underscores the significance of preserving β cells' inter‐organelle communications.

## 6 Conclusions

Mitochondrial homeostasis is pivotal for maintaining pancreatic β cell function, and its disruption plays a central role in the pathogenesis of diabetes. This review elucidates the complex regulatory mechanisms underlying MQC, including biogenesis, dynamics, mitophagy, and proteostasis, all of which are crucial for β cell survival and insulin secretion. Under diabetic conditions, metabolic stressors such as hyperglycemia and lipotoxicity compromise these processes, leading to mitochondrial dysfunction, oxidative stress, and apoptosis. Furthermore, dysregulated inter‐organelle communication, particularly between mitochondria and the endoplasmic reticulum, exacerbates β cell failure. Emerging therapeutic strategies targeting mitochondrial pathways, such as restoring autophagic flux, modulating calcium signaling, and enhancing metabolic reprogramming, provide promising approaches for preserving β cell function. Some of the commercially available GLP1‐RAs and or pre‐clinical glucokinase activator have been shown to conditionally regulate mitochondrial homeostasis in β cells, thereby improving pancreatic islet function.

It is noteworthy that DRAK2 represents a promising therapeutic target for diabetes. Inhibition of DRAK2 activity has been demonstrated to improve mitochondrial quality via the regulation of autophagy in pancreatic β cells. However, further investigation is required to validate these strategies in clinical settings. Bridging the gap between basic mitochondrial biology and translational applications holds the key to developing effective treatments for diabetes, addressing the global challenge posed by this prevalent metabolic disorder.

## Author Contributions

Ruihan Li and Bingqian Zhang undertook the majority of the literature research and organizational tasks, while Yuxin Zhang and Yang Yang contributed to a portion of the literature review and data compilation. Yuting Lu was primarily responsible for the overall structure and drafting of the manuscript. Jingya Li was responsible for the overall conceptualization and final polishing of the entire article.

## Funding

This work was supported by Noncommunicable Chronic Diseases‐National Science and Technology Major Project (2024ZD0523100), National Natural Science Foundation of China (82425058, 82470835, 82200885), and Shanghai Rising‐Star Program (24QA2701700).

## Disclosure

The authors have nothing to report.

## Ethics Statement

The authors have nothing to report.

## Consent

The authors have nothing to report.

## Conflicts of Interest

The authors declare no conflicts of interest.

## Data Availability

Data sharing not applicable to this article as no datasets were generated or analysed during the current study.
